# Haplotypes at the *Phg-2* Locus Are Determining Pathotype-Specificity of Angular Leaf Spot Resistance in Common Bean

**DOI:** 10.3389/fpls.2019.01126

**Published:** 2019-09-12

**Authors:** Michelle M. Nay, Clare M. Mukankusi, Bruno Studer, Bodo Raatz

**Affiliations:** ^1^Molecular Plant Breeding, Institute of Agricultural Sciences, ETH Zurich, Zurich, Switzerland; ^2^Bean Program, International Center for Tropical Agriculture (CIAT), Kampala, Uganda; ^3^Bean Program, International Center for Tropical Agriculture (CIAT), Cali, Colombia

**Keywords:** food security, plant breeding, plant pathology, genome-wide association studies (GWAS), pathotype-specificity, common bean (*Phaseolus vulgaris* L.), angular leaf spot (ALS), *Pseudocercospora griseola*

## Abstract

Angular leaf spot (ALS) is one of the most devastating diseases of common bean (*Phaseolus vulgaris* L.) and causes serious yield losses worldwide. ALS resistance is reportedly pathotype-specific, but little is known about the efficacy of resistance loci against different pathotypes. Here, we report on ALS resistance evaluations of 316 bean lines under greenhouse and field conditions at multiple sites in Colombia and Uganda. Surprisingly, genome-wide association studies revealed only two of the five previously described resistance loci to be significantly associated with ALS resistance. *Phg-2* on chromosome eight was crucial for ALS resistance in all trials, while the resistance locus *Phg-4* on chromosome 4 was effective against one particular pathotype. Further dissection of *Phg-2* uncovered an unprecedented diversity of functional haplotypes for a resistance locus in common bean. DNA sequence-based clustering identified eleven haplotype groups at *Phg-2*. One haplotype group conferred broad-spectrum ALS resistance, six showed pathotype-specific effects, and the remaining seven did not exhibit clear resistance patterns. Our research highlights the importance of ALS pathotype-specificity for durable resistance management strategies in common bean. Molecular markers co-segregating with resistance loci and haplotypes will increase breeding efficiency for ALS resistance and allow to react faster to future changes in pathogen pressure and composition.

## Introduction

Plant diseases can cause substantial loss of crop yields with detrimental effects on food security (Oerke, 2006; Savary et al., 2012). In Latin America and Africa, for example, common bean (*Phaseolus vulgaris* L.) is one of the most important crops and particularly valued for its protein and micronutrient content. However, common bean production is frequently reduced by pathogen attacks with angular leaf spot (ALS), caused by *Pseudocercospora griseola* (Sacc.) Crous and Braun (Crous et al., 2006), being one of the most devastating common bean diseases in the tropics and subtropics. ALS has been reported to cause yield losses of up to 80% (Schwartz et al., 1981; Correa-Victoria et al., 1989; Saettler, 1991; Wortmann et al., 1998; Stenglein et al., 2003; Sartorato, 2004). In the tropics and subtropics, common beans are mostly cultivated by smallholder farmers with limited possibilities to protect their crops from diseases or adverse climatic conditions and, therefore, depend on resistant common bean varieties to maintain stable yields (Schwartz and Pastor-Corrales, 1989).

Common bean germplasm can be divided into two gene pools, the Andean and the Mesoamerican gene pool (Gepts and Debouck, 1991; Mamidi et al., 2013). The latter, genetically more diverse Mesoamerican gene pool has been reported to contain more and stronger ALS resistance sources (Nay et al., 2019). Breeding for ALS resistance is challenged by the high genetic diversity of the pathogen and the recurrent appearance of new *P. griseola* pathotypes (Pastor-Corrales et al., 1998; Busogoro et al., 1999; Mahuku et al., 2002a). To categorize pathotypes, they are tested for their ability to infect six Andean and six Mesoamerican common bean lines with distinct resistance patterns (also referred to as differentials), in order to determine their race (Pastor-Corrales and Jara, 1995; Nay et al., 2019). The ALS pathogen co-evolved within the two common bean gene pools into Andean races, only causing disease on Andean beans, and Mesoamerican races, showing a higher specificity for Mesoamerican beans but also attacking beans of the Andean gene pool (Gepts and Debouck, 1991; Guzmán et al., 1995; Pastor-Corrales et al., 1998; Mamidi et al., 2013; Schmutz et al., 2014). Resistance in common bean has been reported to be pathotype-specific with large differences of the effectiveness in different locations and continents (Pastor-Corrales and Jara, 1995; Pastor-Corrales et al., 1998; Mahuku et al., 2002b; Silva et al., 2008).

Previous ALS resistance studies defined five repeatedly characterized resistance loci, in addition to several minor resistance sources (reviewed in Nay et al., 2019): *Phg-1* was found in the line AND 277, closely linked to the anthracnosis resistance locus *Co-1^4^* at the lower end of chromosome (Chr) 1 (Carvalho et al., 1998; Gonçalves-Vidigal et al., 2011). *Phg-2* was found on Chr 8 in the Mesoamerican lines Mexico 54, with potential resistant alleles in Cornell 49–424, BAT 332, MAR 2, G10474, and G10909 (Sartorato et al., 1999; Ferreira et al., 2000; Nietsche et al., 2000; Caixeta et al., 2003; Mahuku et al., 2004; Mahuku et al., 2011). The *Phg-3* locus was found in Ouro Negro on the lower arm of Chr 4 and *Phg-4* in G5686 on the upper arm (Corrêa et al., 2001; Mahuku et al., 2009; Keller et al., 2015). *Phg-5* was found in the lines CAL 143 and G5686 on Chr 10 (Oblessuc et al., 2012; Keller et al., 2015). Besides these well-characterized major resistance loci, indications for quantitative resistance were reported (Teixeira et al., 2005; Oblessuc et al., 2012; Keller et al., 2015; Bassi et al., 2017).

All the above-mentioned studies were conducted in bi-parental mapping populations, limiting the allelic diversity in the population to the two parental alleles. The establishment of such mapping populations is laborious, and the resistance loci found in such experiments may only be effective in the original background due to epistatic effects (Holland, 2004; Kumar et al., 2014). In addition, bi-parental mapping studies were often tested for ALS resistance with a single pathotype or at a single field location, even though the pathotype-specific resistance reaction of *P. griseola* is well described (Pastor-Corrales et al., 1998; Corrêa et al., 2001; Mahuku et al., 2002b; Mahuku et al., 2009; Gonçalves-Vidigal et al., 2011; Mahuku et al., 2011; Oblessuc et al., 2012; Ddamulira et al., 2014; Bassi et al., 2017). Hence, little is known about the range of effectiveness and the interaction of different ALS resistance loci in common bean in different environments with possibly different pathotypes. Furthermore, all previous mapping studies were conducted with Latin American pathotypes, and it is unknown whether the same resistance loci are effective against pathotypes from Africa.

Genome-wide association studies (GWAS) in panels specifically assembled to contain breeding germplasm with phenotypic variability for the trait of interest can overcome the above-mentioned limitations of bi-parental mapping populations. This type of analysis became possible through technological advancements, particularly in next generation sequencing, which allows to genotype hundreds of individuals at a sufficiently high marker density to cover the linkage disequilibrium blocks and to find trait-specific single nucleotide polymorphisms (SNPs) for breeding. By testing a diversity panel with different pathotypes, GWAS enables the identification of pathotype-specific resistance loci as has been recently demonstrated for anthracnose (Zuiderveen et al., 2016).

The main objective of this study was to gain a broader understanding of ALS resistance sources, the resistance loci they contain, and their effectiveness against different pathotypes on two continents. Specifically, we aimed at i) assembling a panel consisting of the currently available ALS resistance sources, ii) evaluating its resistance against multiple ALS pathotypes under greenhouse and field conditions, and iii) identifying pathotype- and field location-specific resistance loci and haplotypes through genotyping by sequencing (GBS) and GWAS.

## Materials and Methods

### Plant Material

An association mapping panel of 316 common bean lines, named extended BALSIT (extBALSIT), was used for ALS resistance evaluations and GWAS. ExtBALSIT included the Bean ALS International Trial (BALSIT) panel consisting of 55 lines, complemented with previously characterized resistance sources (Carvalho et al., 1998; Sartorato et al., 1999; Ferreira et al., 2000; Nietsche et al., 2000; Corrêa et al., 2001; Caixeta et al., 2003; Mahuku et al., 2003; Mahuku et al., 2004; Mahuku et al., 2009; Gonçalves-Vidigal et al., 2011; Mahuku et al., 2011; Oblessuc et al., 2012; Keller et al., 2015), CIAT breeding material with phenotypic variability for ALS response and susceptible checks. The panel included 124 large-seeded Andean beans, 129 small-seeded Mesoamerican, and 63 lines from inter-gene pool crosses. The 316 common bean lines of the extBALSIT panel were multiplied, out of which 264 lines received phytosanitary certificates and were shipped from Colombia to Uganda for ALS-resistance evaluation.

### Evaluation of Angular Leaf Spot Disease Resistance

The extBALSIT panel was evaluated for ALS resistance in the greenhouse with single-spore *P. griseola* isolates and in the field with mixes of isolates. Highly pathogenic Mesoamerican and Andean races were chosen for the greenhouse experiments. Isolates belonging to races 63–63, 63–47, 30–0, and 13–63 were used in Colombia and race 61–63 in Uganda. In the field, inoculations were conducted with pathogen isolates previously collected at the respective field sites in Colombia and different districts in Uganda (**Supplementary Table 1**). Disease severity was evaluated with the CIAT standard scale ranging from 1 (no disease symptoms) to 9 (very severe disease symptoms and defoliation) (van Schoonhoven and Pastor-Corrales, 1987).

Greenhouse experiments were conducted at CIAT headquarters in Colombia (Cali) and at CIAT in Uganda (Kawanda). Three and five seeds of each common bean line were planted per pot under well-watered conditions in Colombia and Uganda, respectively. In Colombia, primary leaves were treated with Elosal (Bayer Crop Science, Monheim am Rhein, Germany) eight days after sowing, to prevent powdery mildew infections, and urea was added before inoculations. For each pathotype, two replicates in time were screened with each replicate containing one pot per line of the extBALSIT panel. Pathogen isolates were grown in V_8_ medium (Castellanos et al., 2016) for 8–20 days before inoculation, depending on growth rate of the isolate. Inoculum was prepared according to the CIAT manual (Castellanos et al., 2016) and spray-inoculated on trifoliate leaves of 17-day-old plants in Colombia, and 21-day-old plants in Uganda. After inoculation, plants were transferred to a humidity chamber for four days in Colombia, while in Uganda, they were covered with a plastic bag for three days to increase humidity. Ten days after inoculation, plant disease scores were evaluated four times within a week, usually on days 10, 12, 14, and 17. Because of the slow disease progression in Uganda, an additional evaluation was conducted 21 days after inoculation.

Field experiments were conducted during the rainy season in October 2016 and 2017 in Darien (N3 53’31’’ W76 31’0,’’ 1,491 m a.s.l.) and Quilichao (N3 04’22” W76 29’55,” 991 m a.s.l.), Colombia, and in May 2018 in Kawanda (N0 24’11” E32 31’54,” 1,178 m a.s.l.), Uganda. Common bean lines were evaluated as single rows in Colombia and in a randomized complete block design with two replicates in Uganda. The rows measured 2.5–3 m in Colombia and 5 m in Uganda, the distance between rows measured 0.6 m, and seeds were sown with a density of 10 seeds/m. Susceptible and resistant checks were added every eight rows, and a border of susceptible checks was planted to favor spread of the disease. Plants were inoculated three times in a weekly interval using a backpack sprayer, starting approximately 20 days after planting when the third trifoliate leaf of most plants was fully extended (stage V4, according to van Schoonhoven and Pastor-Corrales (1987)). ALS symptoms on leaves were also evaluated three times in a weekly interval and started at the appearance of the first disease symptoms approximately 40 days after inoculation. Pods were evaluated at the mid-pod fill stage, approximately 3 weeks after the last foliar evaluation (exact dates are given in **Supplementary Table 2**). Phenotypic data of the extBALSIT panel is available on dataverse.org (https://doi.org/10.7910/DVN/U2BAWN).

Inoculum was prepared according to Castellanos et al. (2016), as a mixture of five, six, and five single-spore pathogen isolates in Darien, Quilichao, and Kawanda, respectively (**Supplementary Table 1**). The isolates in Uganda did not sporulate well and a precise adjustment of the spore concentration was not possible. Therefore, fungal mycelium of 70 petri dishes was scraped off and diluted in water for the first inoculation and 35 petri dishes for the subsequent inoculations.

### DNA Extraction and Genotyping

For genotyping, three emerging trifoliate leaves were sampled and used for DNA extraction following a urea–phenol–chloroform–isoamylalcohol protocol reported by Chen et al. (1992). DNA quality was checked by agarose gel electrophoresis and quantified by absorption of fluoresce using PicoGreen to stain double stranded DNA (Molecular Probes Inc., Eugene OR, USA). The common bean lines of the extBALSIT panel were subjected to GBS according to Elshire et al. (2011) with the following modifications: adaptor concentrations were 6 ng/μl, digestion per reaction was conducted with 0.5 μl restriction enzyme ApeKI (50 U/μl, New England Biolabs [NEB], Ipswich MA, USA), ligation with 0.5 μl ligase (20 U/μl, Promega, Madison WI, USA) and 3 μl buffer per sample, filled up with ddH_2_O to reach the target reaction volume. After adapter ligation, the 96 samples were pooled and cleaned with a PCR Clean-Up System (Promega), according to the manufacturer’s protocol. For each pool, PCR was conducted in duplicate and merged afterwards. Each PCR reaction with a total volume of 50 μl contained 1x buffer (10 mM Tris-HCl pH 8.8, 50 mM KCl, 0.8% [v/v] Nonidet P40 [Fermentas, Waltham MA, USA]), 2 mM MgCl_2_, 0.1% bovine serum albumin, 1% polyvinylpyrrolidone, 0.016 μM of each primer, 0.4 mM dNTP, 0.3 μl TAQ polymerase (Sigma-Aldrich, St. Louis MN, USA), and 2 μl DNA template. Primers used for amplification were the following: forward PCR_Primer1_Short: AATGATACGGCGACCACCGAGATCTACACTCTTTCCC TACACGACGC and reverse PCR_Primer2.1.i7: AAGCAG AAGACGGCATACGAGATGTCGATTGTGACTGGA GTTCAGATGTGTG. Each library containing 96 individually barcoded genotypes was sequenced by 150 bp single end sequencing on a single lane of the Illumina HiSeq Instrument (Illumina, San Diego CA, USA) at Hudson Alpha sequencing facility (Huntsville AL, USA). For SNP calling, the NGSEP pipeline (Perea et al., 2016) was used with the following quality criteria: a minimum quality score of Q40, scores in at least 220 of the 316 common bean lines, a minor allele frequency exceeding 5%, and a heterozygosity rate below 6%. Subsequently, heterozygous data points were removed. Genomic positions of SNPs and candidate genes were inferred according to the v2.1 of the *P. vulgaris* reference genome (available at https://phytozome.jgi.doe.gov, accessed 11. Nov. 2018). Genotypic information of the extBALSIT panel is available on dataverse.org (https://doi.org/10.7910/DVN/U2BAWN).

### Genome-Wide Association Studies

Genotype to phenotype associations were identified with TASSEL 5 (Bradbury et al., 2007). For greenhouse and field trials, mean ALS scores from the last evaluation of the trial were used. A mixed linear model was implemented using principal component analysis (PCA) with the first two principal components to correct for population structure and the K matrix to correct for kinship (Bradbury et al., 2007). Within TASSEL, the kinship was calculated using the centered identity-by-state (IBS) method, P3D was implemented for variance component analysis, and no compression was used (Zhang et al., 2010; Endelman and Jannink, 2012). The significance threshold was adjusted with the Bonferroni correction. TASSEL output and phenotypic data were analyzed and plotted using RStudio (version 3.4.4) with the packages qqman, ggplot2, reshape2, and psych (R Core Team, 2008).

### Haplotype Analysis at the *Phg-2* Locus

In order to group the haplotypes at the *Phg-2* locus on Chr 8, SNPs located in the interval of significant associations (i.e., from position 61,150,549–62,934,224 bp in the reference genome sequence) were clustered using a hierarchical clustering method implemented in R. The 276 common bean lines with less than 50% missing SNP data in the interval were retained for analysis. The genotype matrix was translated to numeric values, Euclidian distance between the common bean lines calculated and hierarchical clustering according to the Ward.D2 method was performed (Ward, 1963; Murtagh and Legendre, 2014). The resulting dendrogram was cut to group the haplotypes into eleven groups. The haplotype groups were named Andean or Mesoamerican, according to the gene pool of the lines from which the haplotypes originated. To evaluate the effect of the haplotypes, the disease scores of each haplotype for each experiment were plotted in R.

## Results

### Angular Leaf Spot Resistance Is Highly Location- and Pathotype-Specific

**Figure 1:**
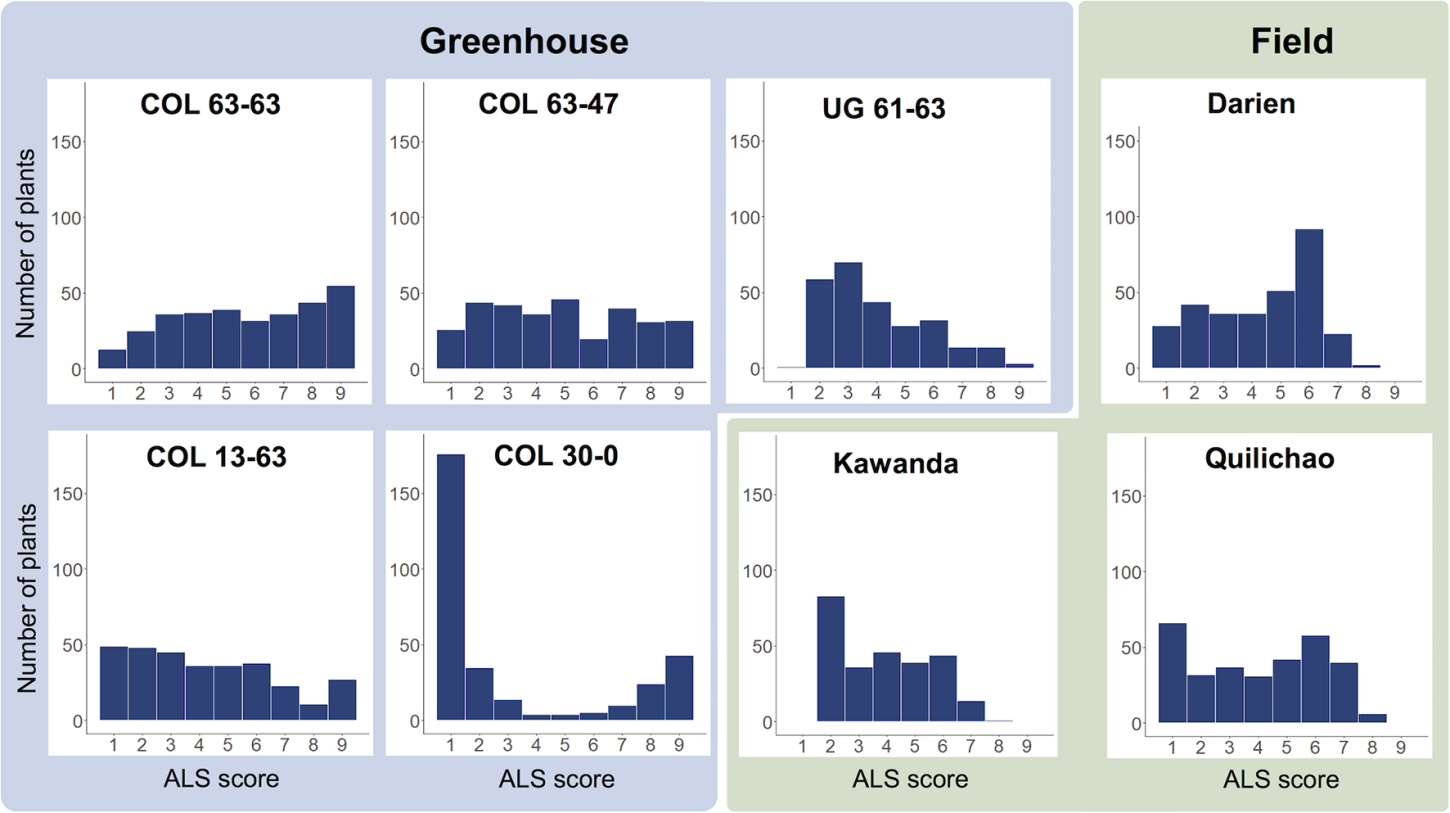
Frequency distributions of disease scores for angular leaf spot (ALS), evaluated in greenhouse (blue) and field trials (green) using the extBALSIT panel containing 316 common bean lines. ALS was scored on a scale from 1 to 9, where 1 is resistant and 9 is highly susceptible. The greenhouse trials were conducted with five different pathotypes, determined by their origin (COL and UG) and race (63–63, 63–47, 61–63, 13–63, and 30–0). Field trials in Colombia (Darien and Quilichao) and Uganda (Kawanda) were inoculated with mixtures of pathotypes previously collected at the corresponding sites. For Darien and Quilichao, the average ALS score from both evaluation years is shown.

Evaluation of the extBALSIT panel for ALS resistance revealed trial-specific frequency distributions of ALS scores (**Figure 1**, **Supplementary Figure 1**). Differences were observed between continents, locations, greenhouse and field experiments, and different pathotypes. For most trials, a continuous distribution of disease scores was found, only in the greenhouse experiment with pathotype COL 30–0, the histogram clearly differentiated resistant and susceptible lines, indicating major gene resistance. Twenty-seven lines were found resistant (ALS score ≤3 on a 1 to 9 scale) in all 6 trials conducted in Colombia, 43 were resistant in the 2 trials conducted in Uganda, and 2 (AAB 8–2, G6727) were resistant in all experiments. The differences between the continents were also notable: of the 46 most resistant lines in Colombia (average ALS leaf score over all experiments ≤3), only 15 had an average score of ≤3 against the Ugandan pathotypes tested.

Out of the 55 pairwise correlations between phenotypic data of the trials, 43 (78%) were significant (Pearson correlation, *P* < 0.05), ranging from 0.12 to 0.73 (**Supplementary Figure 2**). Highest correlations were observed between the replicates of the field experiment in Kawanda, Uganda, and the comparison of field data between years in Darien and Quilichao, Colombia (**Supplementary Table 3**).

### Genome-Wide Association Studies Confirm ALS Resistance Loci on Chromosomes 4 and 8 of Common Bean

Genotyping by sequencing of the extBALSIT panel revealed 22,765 high-quality SNPs distributed over the eleven choromosomes of common bean (**Supplementary Figure 3**). The population structure of the extBALSIT panel was analyzed with PCA, on the basis of the SNP marker data (**Supplementary Figure 4**). The first PC explained 45% of the genotypic variance and clearly distinguished Andean and Mesoamerican lines, with lines that originated from inter-gene pool crosses clustering between them. The second PC explained 4% and distinguished lines that originated from a cross between G10474 and G5687 (referred to as RAI lines) from the remaining inter-gene pool crosses. The second PC further separated the Mesoamerican lines G10613, G10474, G10909, G18970, G855, Mexico 54, G1805, Flor de Mayo, MAR 2, and G5653. The first six of these accessions were collected in Guatemala or neighboring Oaxaca and likely belong to the highly ALS-resistant subpopulation previously characterized in Guatemala (Beebe et al., 2000; Mahuku et al., 2003; CIAT Genebank, 2018; Lobaton et al., 2018).

**Figure 2:**
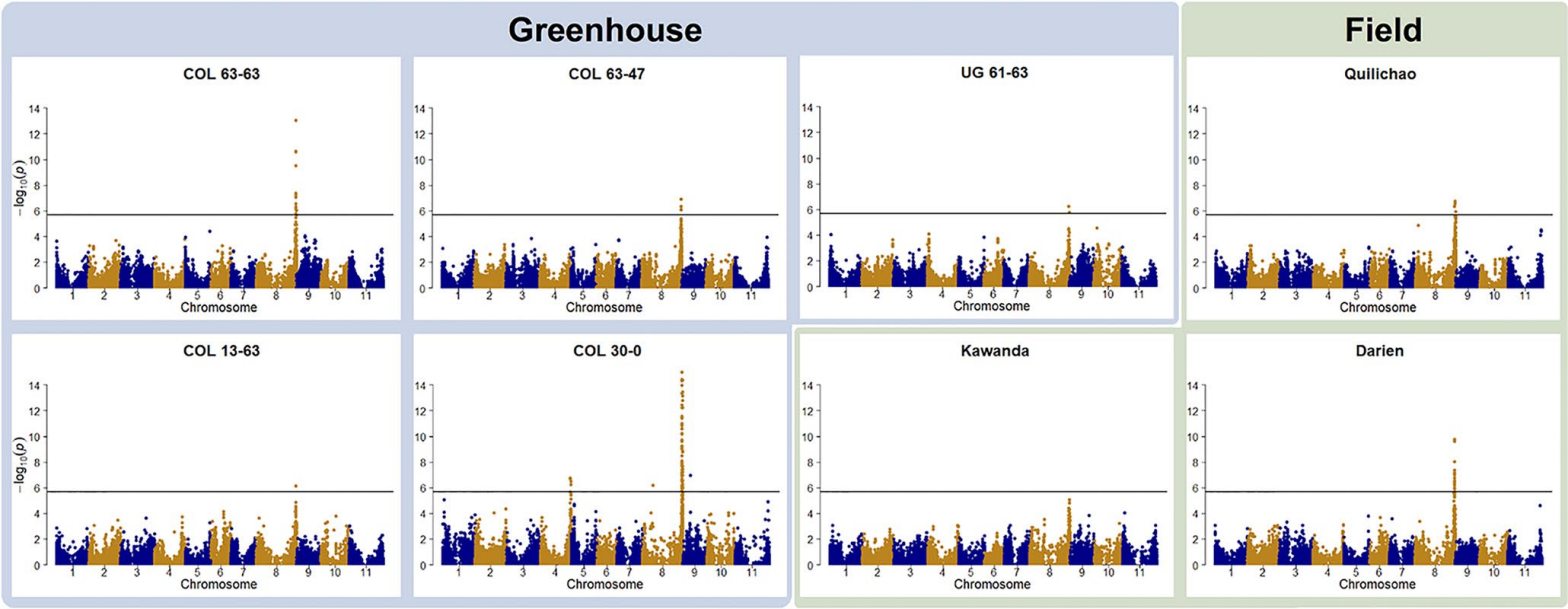
Manhattan plots of the genome-wide association studies (GWAS) for angular leaf spot (ALS) resistance in the extBALSIT panel. The greenhouse trials were conducted with five different pathotypes, determined by their origin (COL and UG) and race (63–63, 63–47, 61–63, 13–63, and 30–0). Field trials in Colombia (Darien and Quilichao) and Uganda (Kawanda) were inoculated with mixtures of pathotypes, previously collected at the corresponding sites. On the x-axis, the genomic position of the markers is given. On the y-axis, the negative logarithm to the base 10 of the *P*-value, representing the significance value, is given. In order to correct for multiple testing, the significance threshold was adjusted through the Bonferroni method, and the new significance threshold is depicted by the black horizontal line.

Genotype to phenotype associations were investigated by GWAS. In all but one trial, foliar ALS resistance was significantly associated with a region on Chr 8 (**Figure 2**). For the field trial in Uganda (Kawanda), a peak is clearly visible in the Manhattan plot, but it is not passing the stringent Bonferroni threshold. Manhattan plots indicate the same resistance locus on Chr 8 to be effective in Colombia as well as in Uganda. The interval where significant associations were found in this study on Chr 8 coincides with the genomic region where molecular markers linked to the *Phg-2* resistance locus in the common bean line Mexico 54 and G10474 were found (Sartorato et al., 1999; Sartorato et al., 2000; Gil et al., 2019), hence, it will be referred to as the *Phg-2* locus. GWAS analyses of ALS symptoms on pods at one of the field locations in Colombia (Darien) resulted in the same resistance locus on Chr 8. Pod evaluations at the other field locations in Colombia (Quilichao) and Uganda (Kawanda), where phenotypic variability was low, did not result in significant associations to markers in the GWAS analysis (**Supplementary Figure 5**). In addition to the predominant signal on Chr 8, another resistance locus on Chr 4 was effective against the pathotype COL 30–0. This resistance locus coincided with the mapping interval of the *Phg-4* locus (Keller et al., 2015).

Over all experiments, significantly associated SNPs were found in the interval spanning 61,150,549–62,934,224 bp (total length of 1,784 kbp) on Chr 8 and 46,703,147–46,934,061 bp (total length of 231 kbp) on Chr 4. In the interval on Chr 8, 265 annotated genes were identified, of which two (Phvul.008G284500, Phvul.008G285300) were NB-ARC domain-containing disease resistance genes (PF00931), another 2 (Phvul.008G267600, Phvul.008G267700) were of the TIR-NBS-LRR class (PF13676, PF01582), and 20 were containing leucine-rich repeats. On Chr 4, 28 annotated genes were found in the interval, but no putative resistance genes were among them. Significant SNPs on Chr 8 explained highest percentages of phenotypic variance, between 8.6–31.4%, in line with the very dominant role of this resistance locus seen in these experiments. Markers associated with the resistance locus on Chr 4 explained 9.3–11.4% of the variance.

### Haplotypes of the Resistance Locus on Chromosome 8 Explain ALS Pathotype-Specificity

**Figure 3:**
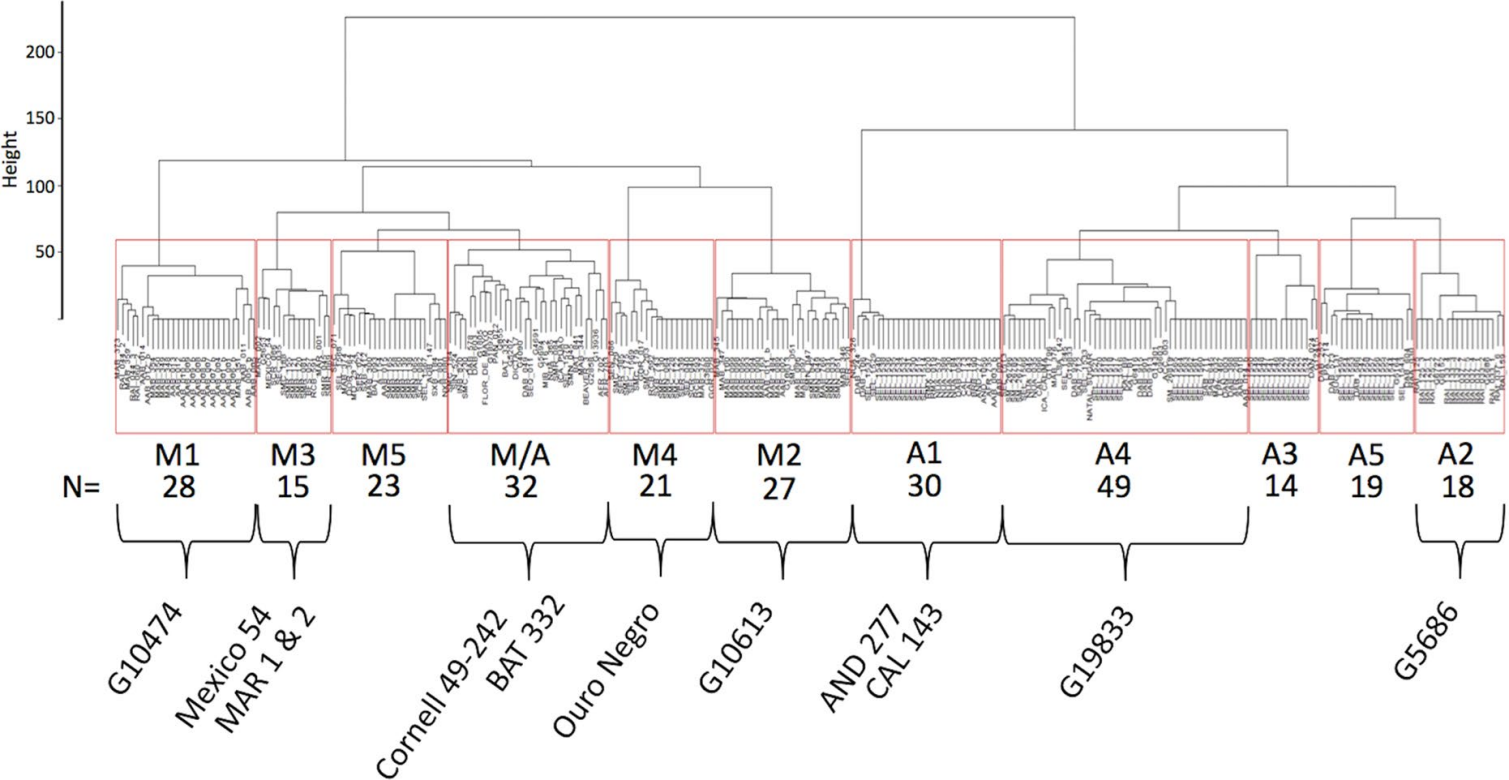
Dendrogram of hierarchical clustering at the *Phg-2* locus. The common bean lines of the extBALSIT panel were clustered according to similarity of their SNP data in the 61.15–62.93 Mbp interval on Chr 8 and divided into eleven haplotype groups. Haplotype groups were named according to the gene pool of the lines (M, Mesoamerican; A, Andean; and M/A = mixed) and numbered. Below the haplotype names, the number of common bean lines in each haplotype group is given and well known ALS resistant common bean lines as well as the reference genome line (G19833) contained in the haplotype groups are indicated. On the y-axis, the Euclidian distance between clusters is shown.

**Figure 4:**
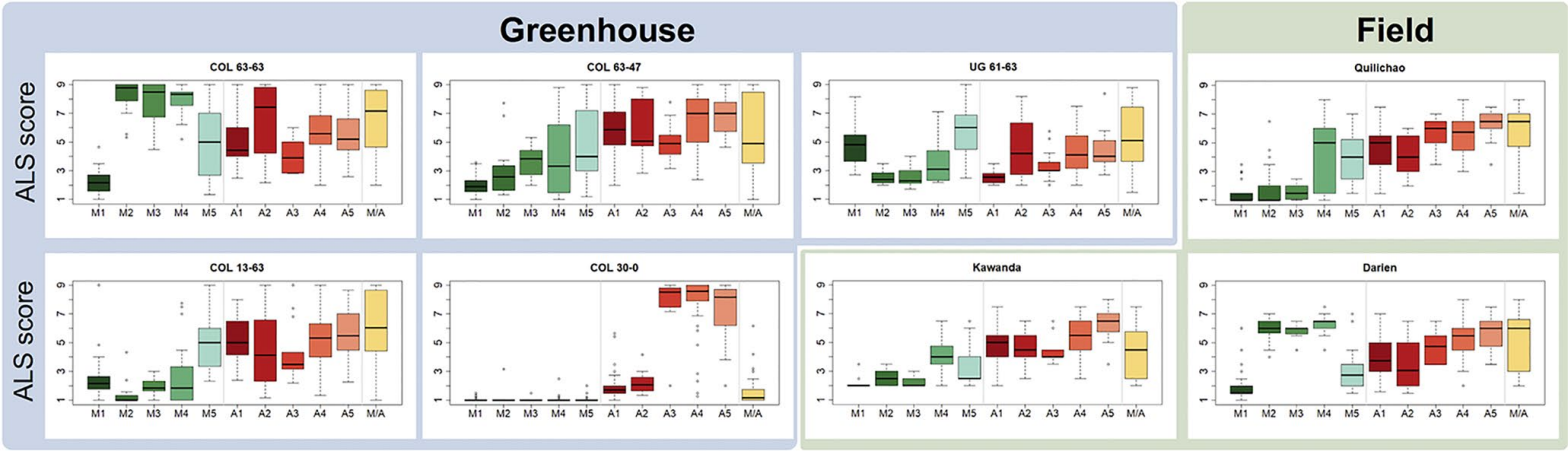
Haplotype groups at the *Phg-2* locus and their ALS response, as evaluated in greenhouse and field trials using the extBALSIT panel. For each trial, the ALS response, scored on a scale from 1 (resistant) to 9 (susceptible), is shown for each of the eleven haplotype groups.

Haplotypes at the *Phg-2* locus, identified through cluster analysis of the SNP data in the *Phg-2* region, were categorized into eleven groups (M1 to M5, M/A, A1 to A5, **Figure 3**) and associated with trial-specific ALS resistance scores (**Figure 4**, **Supplementary Figure 6**). The haplotype groups M1 to M5, originating from the Mesoamerican gene pool, were resistant against the pathotype COL 30–0, as indicated by its race code. Common bean lines from the Mesoamerican haplotype group M1 were resistant in nearly all experiments but showed intermediate resistance in the trial with the Ugandan pathotype UG 61–63. Lines from the haplotype groups M2 and M3 were resistant against COL 14–63, UG 61–63, and the pathotypes present in the field in Quilichao and Kawanda but were susceptible to pathotypes present in the field in Darien and the most aggressive race COL 63–63. Lines from the haplotype group M4 showed increased resistance against UG 61–63 and COL 13–63 but were less effective compared to M2 and M3. Lines from the haplotype group M5 were largely resistant against pathogen races in Darien and Kawanda, but no clear trend was observed in the other experiments.

Andean haplotype groups at the *Phg-2* locus were mostly associated with susceptibility to ALS. A1 and A2 only displayed effective resistance against COL 30–0, and A1 and A3 appeared resistant against UG 61–63. Lines from the haplotype groups A4, A5, and M/A were mostly susceptible in all experiments. The haplotypes at the *Phg-2* locus were able to explain a much larger fraction of the total phenotypic variability in ALS resistance (R^2^ = 0.40 – 0.85, **Supplementary Table 4**) compared to significant single SNP markers.

### Haplotype-Specific SNPs to Advance Resistance Breeding by Marker-Assisted Selection

**Figure 5:**
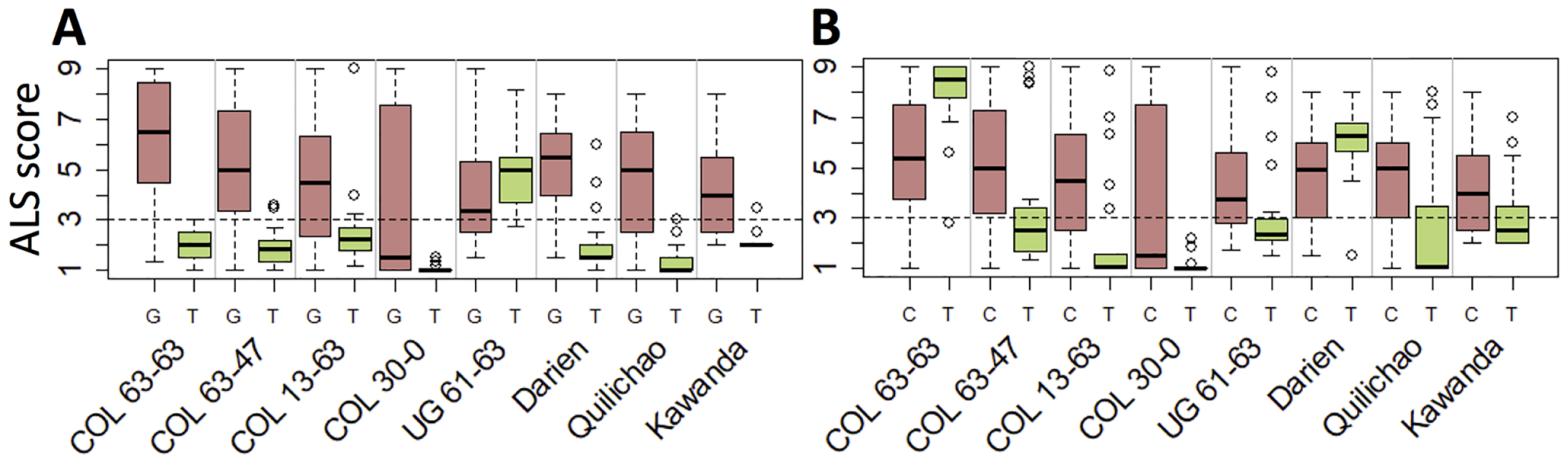
Candidate SNPs for marker-assisted selection of *Phg-2* haplotypes. Shown is the phenotypic distribution of ALS scores of the two alleles at the SNPs, which are specific for the functional haplotypes M1 **(A)** and M2 **(B)**. The SNPs on chromosome 8 at position 61,901,182 bp and 62,188,623 bp of the Pv2.1 reference genome that co-segregated with the haplotype groups M1 and M2, respectively, were used. On the y-axis, ALS response scored on a scale from 1 to 9 is shown, whereas scores below 3 (dashed line) are considered resistant. On the x-axis, greenhouse and field trials are indicated, and for each trial, the ALS-resistance response of the two alleles of the SNP is plotted.

Seven haplotype groups (M1–3, M5, A1–A3) were identified as potentially interesting for breeding because of the resistance they displayed in multiple experiments. For example, the SNP marker specific for M1, the haplotype group associated with strongest resistance against most pathotypes, offers unique opportunities to trace this effective resistance allele in advanced breeding germplasm (**Figure 5A**). Similarly, the SNP markers tagging M2 (**Figure 5B**) and M3 (**Supplementary Table 5**) can be employed for breeding to provide resistance against UG 61–63 (and the region of its occurrence). The SNP markers specific for the Andean haplotype groups A1 and A2 can be used to improve ALS resistance in the Andean gene pool, although their effectiveness is limited to a few pathotypes only. Genomic positions of the SNPs specific for all but one of the seven haplotype groups as well as for the resistance locus on Chr 4 are provided in **Supplementary Table 5**.

## Discussion

This study is the first to thoroughly evaluate the pathotype-specific response of ALS in common bean on the genetic level. Through GWAS in the largest yet assembled diversity panel segregating for ALS resistance, a pathotype-specific resistance locus, likely *Phg-4*, and a broad-spectrum resistance locus coinciding with *Phg-2* were effective against a variety of ALS pathotypes from Colombia and Uganda. For the latter locus, a high haplotype diversity was found, with at least seven different haplotype groups providing resistance in a pathotype-specific manner. Molecular markers specific for resistance loci and haplotype groups will facilitate breeding for pathotype-specific ALS resistance through marker-assisted introgression strategies.

### No Effect of *Phg-1*, *Phg-3*, and *Phg-5* Against the ALS Pathotypes Tested

In common bean, ALS resistance is reportedly controlled by five major resistance loci, named *Phg-1* to *Phg-5* (Souza et al., 2016). Our study revealed a preeminent role of *Phg-2*, representing the unmatched source of resistance in effectively all experiments, while *Phg-1*, *Phg-3*, and *Phg-5* did not appear to be relevant. This is unexpected as the resistance loci *Phg-1* and *Phg-5*, originating from the resistance sources AND 277, CAL 143, and G5686 that were extensively used as progenitors in the CIAT breeding program, were present in the extBALSIT panel at frequencies sufficiently high to be detected by GWAS. Our observation may be a consequence of the strong pathotype-specificity of *P. griseola* and the differences in pathotypes prevalent within regions, countries, and continents. Experiments that led to the discovery of *Phg-1* and *Phg-3* were conducted with ALS evaluation protocols comparable to ours using pathotypes of the races 63–23 and 63–39 from Brazil (Gonçalves-Vidigal et al., 2011; Gonçalves-Vidigal et al., 2013). *Phg-5* was discovered in CAL143 using a pathotype of race 0–39 and natural field evaluations in Brazil, and in G5686 using a pathotype of race 31–0 from Colombia (Oblessuc et al., 2012; Oblessuc et al., 2013; Keller et al., 2015). Brazilian pathotypes are known to be very aggressive on the current differentials (Balbi et al., 2009; Nietsche et al., 2001; Sartorato and Alzate-Marin, 2004; Silva et al., 2008), and it is possible that specific resistance genes are effective against these pathotypes. Future experiments should involve resistance evaluations of the extBALSIT panel with additional pathotypes, particularly from Brazil, where the resistance loci *Phg-1, Phg-3*, and *Phg-5* were observed to be effective.

In a similar study on resistance to anthracnose in common bean, an Andean bean diversity panel was tested with eight different pathotypes. In contrast to our study that only revealed a small subset of previously reported ALS resistance loci, GWAS for anthracnose resistance found the majority of the known resistance loci to effectively be involved (Zuiderveen et al., 2016). Our findings undermine the importance of the pathotypes on the efficacy of disease resistance in common bean and call for an increased understanding of the pathogen population structure and virulence to allow prediction of effectiveness of resistance loci. Once the population structure of the pathogen is better known, established GWAS panels can be used to study the pathotype-specificity within and between sub-populations.

### *Phg-2* Is an Important ALS Resistance Locus With Functional Haplotypes From the Mesoamerican and the Andean Background

The *Phg-2* locus is one of the most important ALS resistance locus in common bean and originally described in the Mesoamerican cultivar Mexico 54 (Sartorato et al., 1999; Sartorato et al., 2000). In the meantime, several additional Mesoamerican common bean lines were found to contain ALS resistance, either at or in close proximity to *Phg-2* on Chr 8 (Nietsche et al., 2000; Mahuku et al., 2004; Namayanja et al., 2006; Mahuku et al., 2011). This led to the hypothesis that *Phg-2* originated from the Mesoamerican gene pool, and hence, several breeding efforts aimed at its introgression into the Andean gene pool. Our study revealed that ALS resistance at *Phg-2* can also be found in the Andean gene pool: through cluster analysis on the basis of the genotypic data in the *Phg-2* region, we were able to classify eleven haplotype groups, at least seven of which appeared to be functionally different, leading to distinct patterns of resistance against the tested ALS pathotypes. Not only were resistance- and susceptibility- associated haplotypes in both gene pools, but also within each gene pool different haplotype groups of this resistance locus provided resistance to some, but not all evaluated ALS pathotypes.

### Genetic Determination of Pathotype-Specificity at *Phg-2* on Chromosome 8

The different haplotype groups at *Phg-2* largely explained pathotype-specificity for ALS response. For further understanding of the detailed interaction on the molecular level, the underlying genetic determinants need to be identified. To date, the causal genes of any ALS resistance loci, including *Phg-2*, are yet to be determined. Based on our data, it remains difficult to resolve whether the resistance at *Phg-2* is conferred by an allelic series at one resistance gene or by several resistance genes arrayed in clusters within the haplotype region defined by the significantly associated SNP markers.

Both, resistance gene clusters and allelic series are commonly occurring in plants (Keller et al., 2000). In common bean, several allelic series have been reported for anthracnose resistance, and there were five alleles described of the *Co-1* and *Co-3* loci, three alleles for the *Co-4* locus, and two alleles for the *Co-5* locus (BIC Genetics Committee, 2017). In wheat, up to 17 alleles have been found for the powdery mildew resistance gene *Pm3* that showed different pathotype-specific reactions (Bhullar et al., 2010). The *Pm3* gene encoded for a classical nucleotide binding leucine-rich repeat (NR-LRR) receptor and alleles were highly similar, with usually only single amino acid changes differing between the alleles (Yahiaoui et al., 2009). This pattern is reflecting the evolutionary mechanisms that are promoting the genetic diversification of resistance loci (Ellis et al., 2000; Bergelson et al., 2001).

Although the presence of allelic series may be a plausible explanation for ALS pathotype-specificity, the large extension of the *Phg-2* region, spanning 1.78 Mbp including 265 annotated genes, as well as the pathotype-specific significance peaks at distinct positions within this interval, indicates the involvement of multiple genes. Indeed, the presence of several candidate NB-LRR resistance genes at *Phg-2* in the Andean reference genome and their distinct expression in leaf tissue, as the case with Phvul.008G284500 and Phvul.008G285300 (Phytozome, 2018), strengthens this hypothesis.

While these are probable candidate genes, it should be noted that the most effective haplotype groups at *Phg-2* originated from the Mesoamerican gene pool, while the reference genome used for SNP discovery and gene identification derived from the Andean gene pool (Schmutz et al., 2014). Resistance gene clusters are repetitive arrays of highly similar gene sequences that are often difficult to correctly assemble (Belser et al., 2018). Moreover, they usually differ in the number of repeats between common bean lines and gene pools, and hence, one reference genome might not be fully representative of the structural diversity at resistance loci. In the recent years, novel genome assembly strategies including long-read sequencing technologies have been developed to assemble such regions more accurately. With the increased availability of pan-genomes, it will be possible to take into account even the genetic rearrangements between common bean lines.

### Implications for ALS Resistance Breeding

ALS is one of the most devastating common bean diseases, particularly affecting smallholder farmers in low input agricultural systems. The results are production losses to the poorest, which most depend on the harvest from their fields for food security. Breeding for ALS resistance and other biotic and abiotic stress has been ongoing for a long time in common bean breeding programs of the tropics (Beebe, 2012), but in the future, breeding needs to respond quicker than in the past to assure food security and adequate nutrition. Globalization and the increased human mobility have led to a globalization of plant pathogens and will continue to facilitate the exchange of genetic pathogen diversity (Anderson et al., 2004; Jeger et al., 2011; Fisher et al., 2012; Santini et al., 2018). An additional process that is expected to heavily affect plant pathogen dynamics is climate change. The increased warming and occurrence of extreme weather events will have effects on prevalence and plant-pathogen interactions (Anderson et al., 2004; Elad and Pertot, 2014). In the case of the tropical pathogen ALS, global warming will likely expand its range, and global mobility will lead to a mixing of pathogen populations previously separated by distance. More effective breeding methods are therefore urgently required to develop the varieties that will feed the growing future populations in developing countries. The research presented here will increase breeding efficiency for ALS resistance by providing a screening panel that can be used to find effective resistance loci in different areas. The molecular markers linked to resistance loci and resistance haplotypes will allow development of resistant lines without direct phenotypic screening in the region.

Resistance gene pyramiding is usually the suggested strategy to ensure durable disease resistance for highly virulent pathogens such as *P. griseola* (Pastor-Corrales et al., 1998). The fact that ALS resistance in nearly all trials was conferred by the different haplotypes at *Phg-2* is rendering pyramiding difficult or impossible, depending on whether the causal genes are different genes within a resistance gene cluster or allelic series, respectively. Until the causal genes are known, the haplotype groups with very high effectiveness on both continents, M1 for Colombia and M2 and M3 for Uganda, provide the most sustainable strategy to control ALS by marker-assisted selection in one of the globally most important food security crop. However, given the threat of resistance sources to become inefficient, it is crucial to seek new ALS resistant common bean lines and elucidate the genetics of their resistance (Nay et al., 2019).

## Data Availability

The datasets analyzed and generated for this study can be found on dataverse at https://doi.org/10.7910/DVN/U2BAWN.

## Author Contributions

BR and BS conceived the study. MN conducted the experiments and performed data analysis and interpretation. BR, BS, and CM assisted in the experimental setup and data analysis. MN drafted the manuscript, which was improved by BS, BR, and CM. All authors read and approved the final manuscript.

## Funding

This project was funded by ETH Global, the Sawiris Foundation for Social Development and supported by the Molecular Plant Breeding group of ETH Zurich. We would also like to acknowledge funding support from the Tropical Legumes III – Improving Livelihoods for Smallholder Farmers: Enhanced Grain Legume Productivity and Production in Sub-Saharan Africa and South Asia (OPP1114827) project funded by the Bill & Melinda Gates Foundation.

## Conflict of Interest Statement

The authors declare that the research was conducted in the absence of any commercial or financial relationships that could be construed as a potential conflict of interest.
