# Supplementary material for: Haplotypes at the Phg-2 Locus Are Determining Pathotype-Specificity of Angular Leaf Spot Resistance in Common Bean
Source: Front Plant Sci. 2019 Sep 12;10:1126. doi: 10.3389/fpls.2019.01126 (PMC6753878; doi:10.3389/fpls.2019.01126)
Supplement: Supplementary file 1 [file DataSheet_1.docx]

Supplementary Material

# Supplementary Data

##
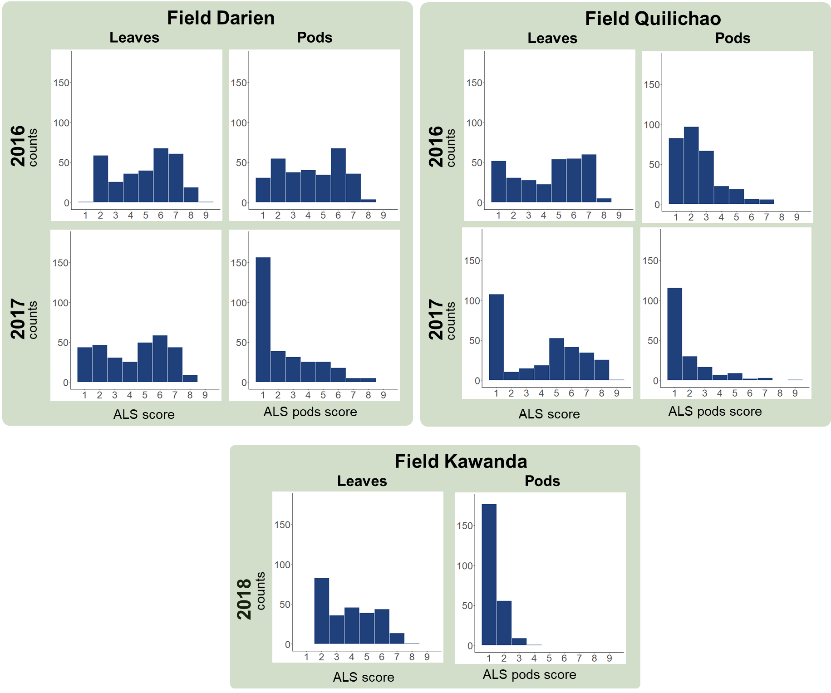

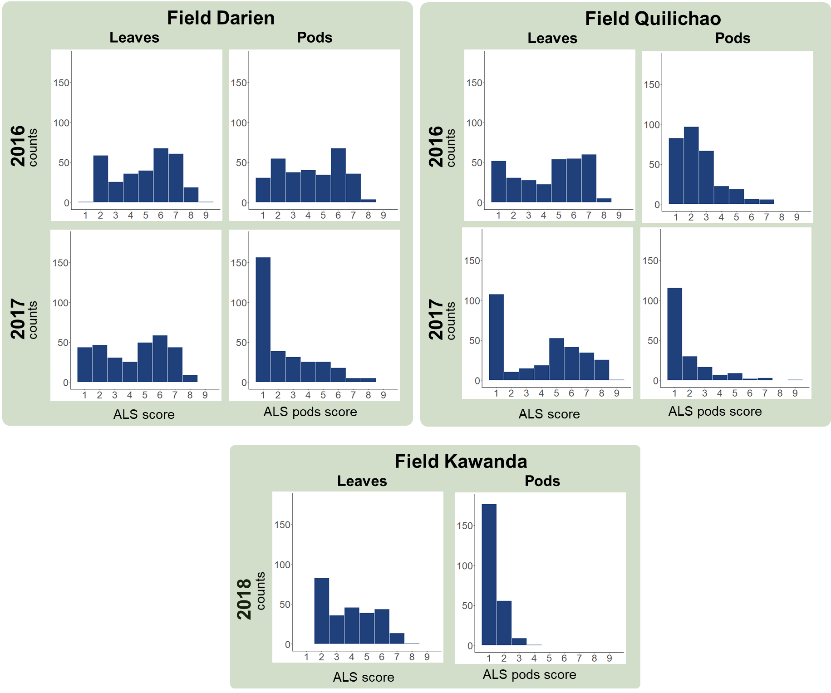
Supplementary Figures

**Supplementary Figure 1**. Frequency distributions of disease scores for angular leaf spot in field experiments using the extBALSIT panel containing 316 common bean lines. Angular leaf spot was scored on leaves and pods on a scale from 1 (resistant) to 9 (highly susceptible). Field trials in Colombia (Darien and Quilichao) and Uganda (Kawanda) were inoculated with mixtures of pathotypes, previously collected at the corresponding sites. Experiments in Colombia were conducted in two different years.


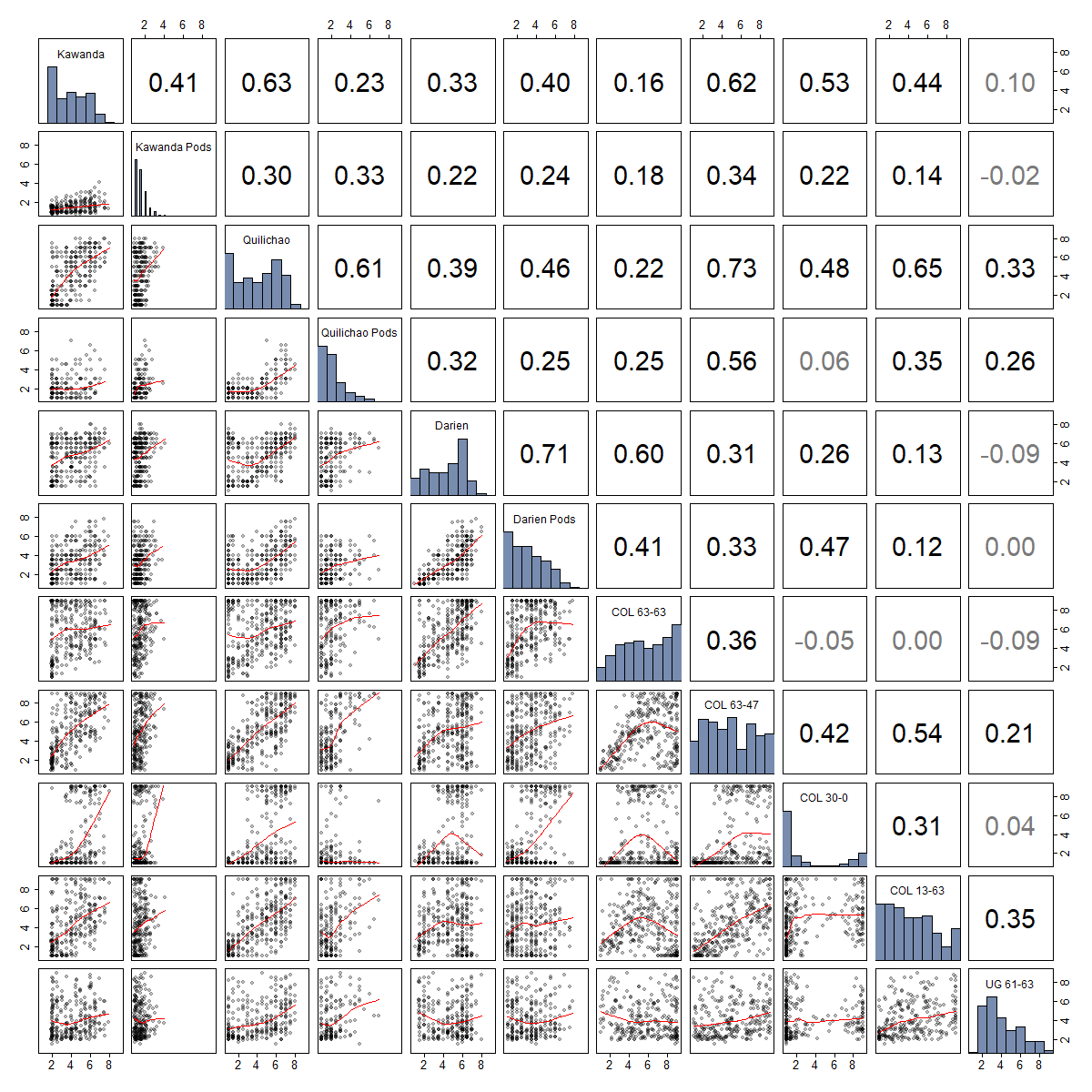


**Supplementary Figure 2.** Correlation matrix of disease scores for angular leaf spot (ALS), evaluated in different greenhouse and field trials using the extBALSIT panel containing 316 common bean lines. Greenhouse trials were conducted with five different pathotypes, determined by their origin (COL and UG) and race (63-63, 63-47, 61-63, 13-63 and 30-0), and ALS scores were taken on leaves. Field trials in Colombia (Darien and Quilichao) and Uganda (Kawanda) were inoculated with mixtures of pathotypes, and ALS scores on leaves and pods were recorded. In the upper diagonal, Pearson correlations between trials are shown as well as their significance. Significant correlations (*P* < 0.05) are shown in black font and non-significant correlations in grey font. In the diagonal, histograms of ALS scores for each trial are given. In the lower diagonal, ALS scores of the two trials are plotted with the red line representing the LOESS (locally estimated scatterplot smoothing) line.


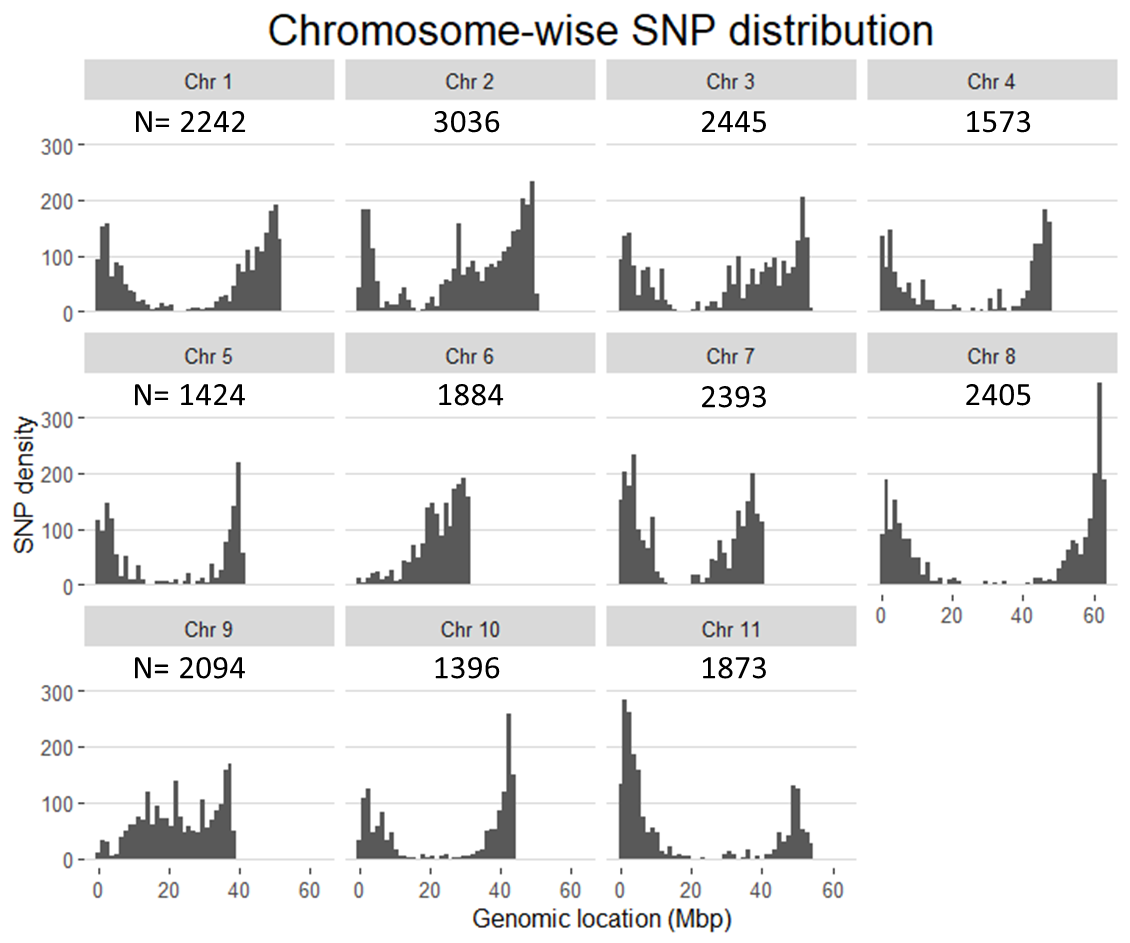


**Supplementary Figure 3.** Distribution of SNP markers in the extBALSIT panel on the eleven chromosomes of the common bean reference genome. Histograms show the SNP density on chromosomes, and the total number of SNPs per chromosome is listed above each histogram. The common bean reference genome v2.1 was used as a reference.


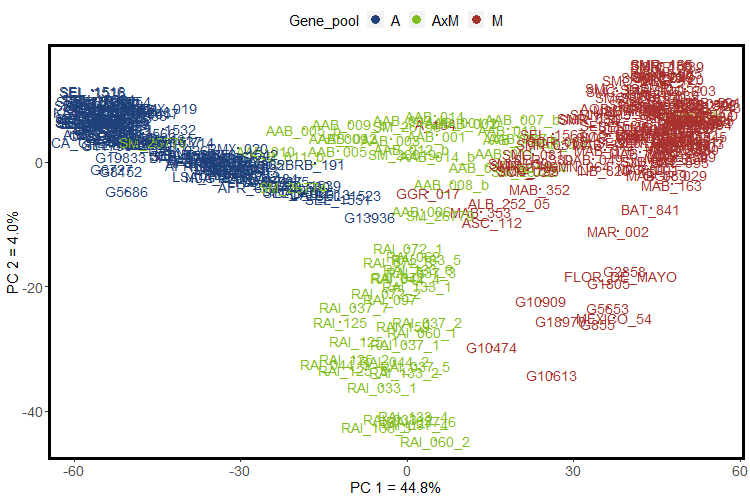


**Supplementary Figure 4.** Population structure of the 316 common bean lines of the extBALSIT panel based on genotypic data. Principal component analysis was conducted using 22,765 SNP markers distributed over the eleven chromosomes. Bean line names are colored according to their gene pool of origin: A (Andean), M (Mesoamerican) and AxM (Inter-gene pool cross).


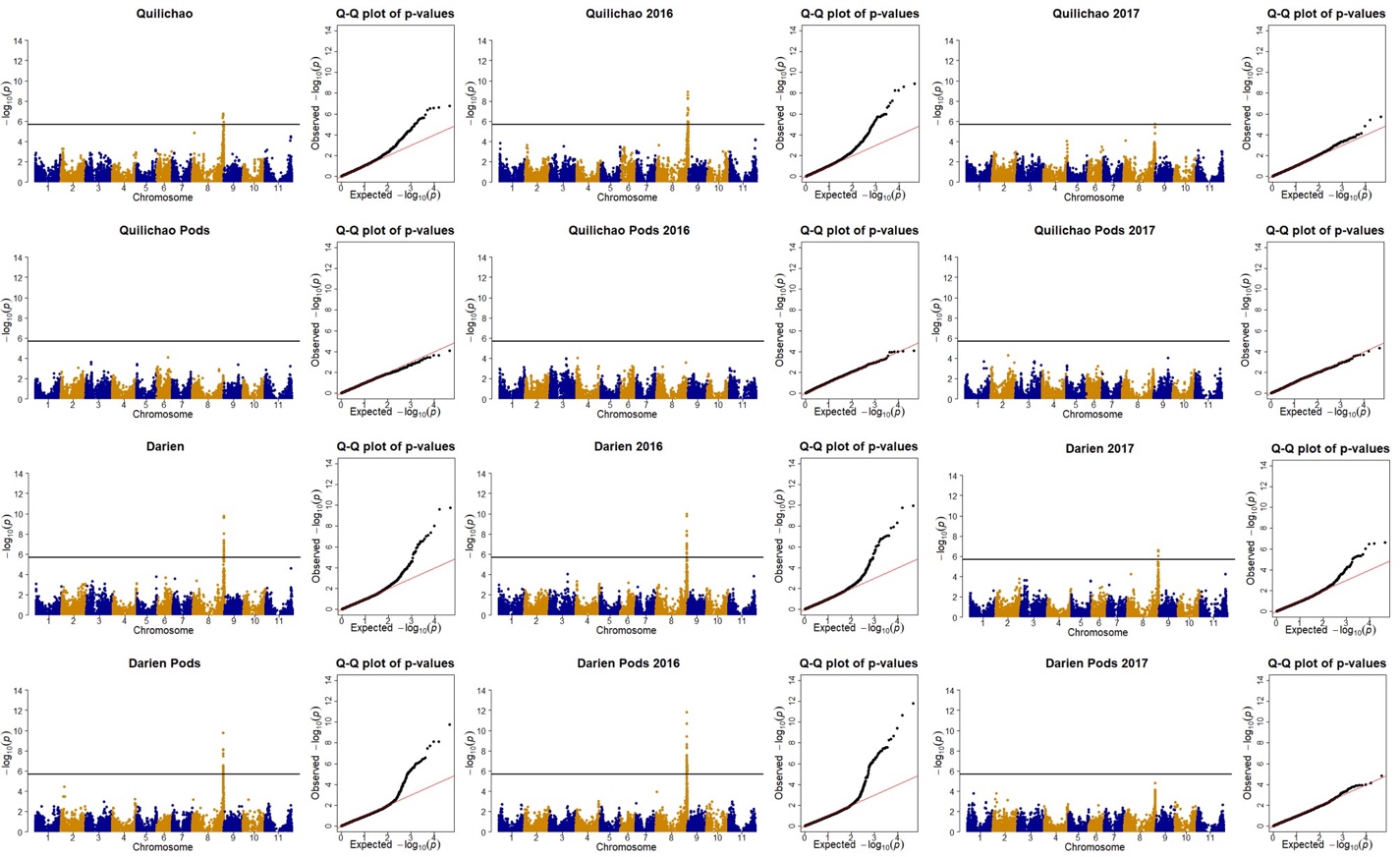


**Supplementary Figure 5.** Comparison of genome-wide association study results for angular leaf spot resistance of field experiments in two years. Manhattan and quantile-quantile (Q-Q) plot are shown for the years 2016 (central column) and 2017 (right column) separately and for the mean over both years (left column). Field trials in Colombia (Darien and Quilichao) were inoculated with mixtures of pathotypes, previously collected at the corresponding sites. On the x-axis, the genomic position of the SNP markers is given in the Manhattan plot and the negative logarithm to the base 10 of the expected *P*-value is given in the Q-Q plot. On the y-axis, the negative logarithm to the base 10 of the *P*-value, representing the significance value, is given. In order to correct for multiple testing, the significance threshold was adjusted through the Bonferroni method and the new significance threshold is depicted by the black horizontal line.


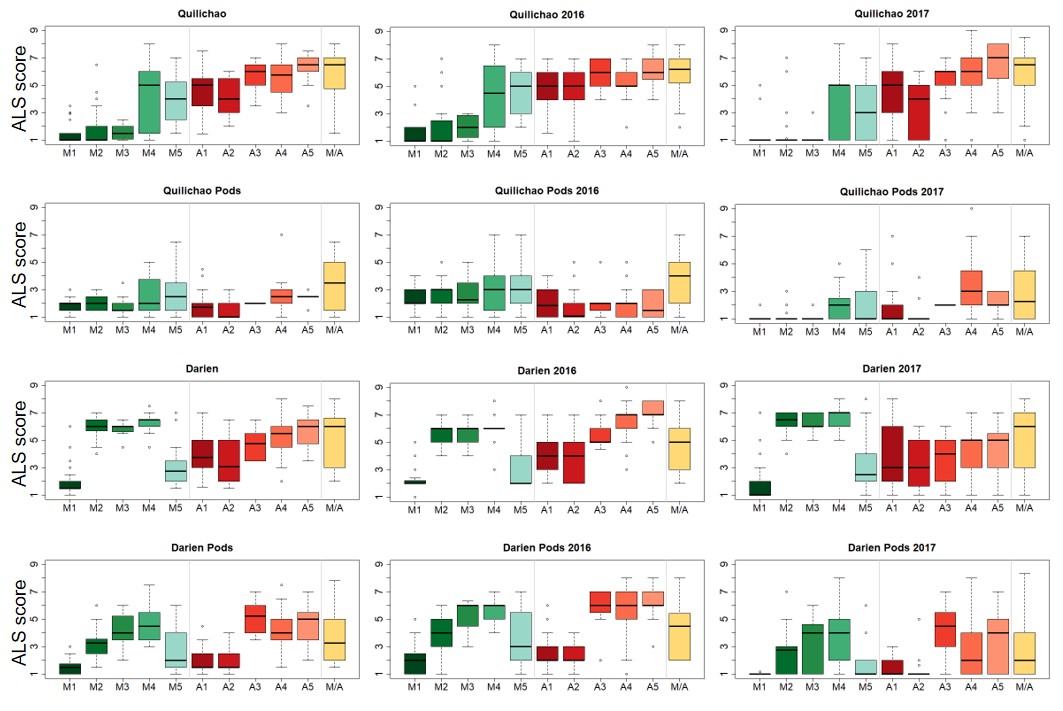


**Supplementary Figure 6.** Haplotype-specific angular leaf spot responses of the extBALSIT panel in field trials conducted in two years. Common bean lines with genetic similarity at the *Phg-2* locus were grouped and haplotype groups named Mesoamerican (M1-3), Andean (A1-4) and mixed (M/A) based on their gene pool origin. Experiments in Colombia were conducted in two years and haplotype-specific angular leaf spot responses are shown for the years 2016 (center column) and 2017 (right column) separately. In the left column, the mean over the two years is shown. On the x-axis the haplotype group is given. On the y-axis, the angular leaf spot response scored on a scale from 1 (resistant) to 9 (highly susceptible) is shown.

## Supplementary Tables

**Supplementary Table 1.** *Pseudocercospora griseola* pathotypes used for angular leaf spot resistance evaluation of the extBALSIT panel in greenhouse and field experiments. For each pathotype, the country of origin (COL = Colombia and UG = Uganda), race, isolate number and the collection site is given.

| **Origin and Race** | **Isolate** | **Experiment** | **Collection site** |
| --- | --- | --- | --- |
| COL 63-63 | Pg 347 | Greenhouse | Quilichao |
| COL 63-47 | Pg 431 | Greenhouse | Tenerife |
| COL 30-0 | Pg 447 | Greenhouse | Palmira |
| COL 13-63 | Pg 61 | Greenhouse | Quilichao |
| COL 31-47 | Pg 44 | Field Darien | Darien |
| COL 5-47 | Pg 81 | Field Darien | Darien |
| COL 31-47 | Pg 261 | Field Darien | Darien |
| COL 15-44 | Pg 305 | Field Darien | Darien |
| COL 63-0 | Pg 66 | Field Darien | Darien |
| COL 63-0 | Pg 3 | Field Quilichao | Quilichao |
| COL 7-35 | Pg 15 | Field Quilichao | Quilichao |
| COL 31-55 | Pg 32 | Field Quilichao | Quilichao |
| COL 13-63 | Pg 61 | Field Quilichao | Quilichao |
| COL 15-39 | Pg 318-1 | Field Quilichao | Quilichao |
| COL 31-47 | Pg 254 | Field Quilichao | Popayan |
| UG 61-63 | KA 060 | Greenhouse | Kabale |
| UG 13-13 | KA 045 | Field Kawanda | Kabale |
| UG 0-22^†^ | KA 049A | Field Kawanda | Kabale |
| UG 1-6^†^ | KA 039A | Field Kawanda | Kabale |
| UG 17-23 | MB 026 | Field Kawanda | Mbale |
| UG 1-22^†^ | KIS 70B | Field Kawanda | Kisoro |

^†^ These Ugandan races were evaluated with an incomplete set of
 differentials. Missing were the Andean bean lines Poroto (binary
 value = 2) and Bolon Bayo (4).

**Supplementary Table 2.** Sowing, inoculation and evaluation dates for field experiments conducted to evaluate the resistance of the extBALSIT panel to angular leaf spot in common bean. Given are the dates (and days after sowing in brackets) for each field trial. Plant development was delayed in Uganda (Kawanda) in comparison to Colombia (Darien and Quilichao) because of water limitation during the germination phase. Therefore, inoculation and evaluations were conducted approximately one week later than in Colombia.

| **Location / Year** | **Darien  2016** | **Darien  2017** | **Quilichao 2016** | **Quilichao 2017** | **Kawanda  2018** |
| --- | --- | --- | --- | --- | --- |
| **Sowing date** | 27.10.16 | 27.10.17 | 18.11.16 | 25.10.17 | 22.5.18 |
| **Inoculations**  1^st^  2^nd^  3^rd^ | 18.11.16 (d22)  24.11.16 (d28)  2.12.16 (d36) | 20.11.17 (d24)  27.11.17 (d30)  4.12.17 (d37) | 07.12.16 (d19)  14.12.16 (d26)  21.12.16 (d33) | 17.11.17 (d23)  24.11.17 (d30)  1.12.17 (d37) | 22.7.18 (d31)  29.7.18 (d38)  6.7.18 (d45) |
| **Evaluations**  1^st^  2^nd^  3^rd^ | 2.12.16 (d36)  9.12.16 (d43)  26.12.16 (d60) | 7.12.17 (d41)  15.12.17 (d49)  21.12.17 (d55) | 27.12.16 (d39)  4.1.17 (d47)  13.1.17 (d56) | 7.12.17 (d43)  13.12.17 (d49)  19.12.17 (d55) | 6.7.18 (d45)  13.7.18 (d52)  20.7.18 (d59) |
| **Evaluation Pods** | 11.1.17 (d76) | 9.1.18 (d74) | 31.1.17 (d74) | 12.1.18 (d79) | 10.8.18 (d80) |

**Supplementary Table 3.** Correlations and mean phenotypic values for angular leaf spot resistance of the extBALSIT panel evaluated in different years or replicates. The field trials in Colombia (Darien and Quilichao) were conducted in different years, while the field trial in Uganda (Kawanda) was conducted with two replicates. Greenhouse trials were conducted with five different pathotypes, determined by their origin (COL and UG,) and race (63-63, 63-47, 13-63 and 30-0). For each trial, the mean values and the standard deviation is given. In the last column, the Pearson correlation (*r*) between years and replicates is given.

| **Trial** | **Average** | **Year / Rep 1** | **Year / Rep 2** | **Correlation *r* =** |
| --- | --- | --- | --- | --- |
| Darien | 4.60 ± 1.83 | 4.94 ± 1.95 | 4.26 ± 2.15 | 0.59 |
| Darien Pods | 3.31 ± 1.71 | 4.17 ± 2.00 | 2.44 ± 1.87 | 0.56 |
| Quilichao | 4.22 ± 2.20 | 4.39 ± 2.19 | 3.98 ± 2.55 | 0.72 |
| Quilichao Pods | 2.29 ± 1.27 | 2.48 ± 1.42 | 1.86 ± 1.47 | 0.44 |
| Kawanda | 4.10 ± 1.69 | 3.91 ± 1.85 | 4.29 ± 1.85 | 0.67 |
| Kawanda Pods | 1.51 ± 0.56 | 1.57 ± 0.82 | 1.46 ± 0.68 | 0.13 |
| COL 63-63 | 5.74 ± 2.42 |  |  |  |
| COL 63-47 | 4.92 ± 2.45 |  |  |  |
| COL 13-63 | 4.30 ± 2.44 |  |  |  |
| COL 30-0 | 3.25 ± 3.13 |  |  |  |
| UG 61-63 | 4.11 ± 1.82 |  |  |  |

**Supplementary Table 4.** Phenotypic variance for angular leaf spot (ALS) resistance in the extBALSIT panel explained by the *Phg-2* haplotype groups in greenhouse and field trials. Reported here is the coefficient of determination (R^2^) of a linear model with the haplotypes as the predictor and ALS score as target variable.

| **Isolate** | **R^2^** |
| --- | --- |
| COL 63-63 | 0.524 |
| COL 63-47 | 0.403 |
| COL 13-63 | 0.421 |
| COL 30-0 | 0.850 |
| UG 61-63 | 0.351 |
| Darien | 0.511 |
| Quilichao | 0.557 |
| Kawanda | 0.567 |
| Darien Pods | 0.471 |
| Quilichao Pods | 0.220 |
| Kawanda Pods | 0.161 |

**Supplementary Table 5.** Locus- and *Phg-2* haplotype-specific SNP markers for breeding applications. Provided is a non-exhaustive list of SNPs that are locus- or haplotype-specific and represent target SNPs for marker-assisted selection in common bean. The SNP position names consist of the chromosome (Chr) number and the genetic position (pos) on the common bean reference genome v2.1. In the SNP column, the resistant allele is given before the dash and the susceptible allele after.

| **SNP position** | **SNP** | **Allele-specific reaction in ALS trials** |
| --- | --- | --- |
| ***Phg-4* on chromosome 4** | | |
| Chr04pos46703147  Chr04pos46934061  Chr04pos46727398 | G/A  T/C  G/A | 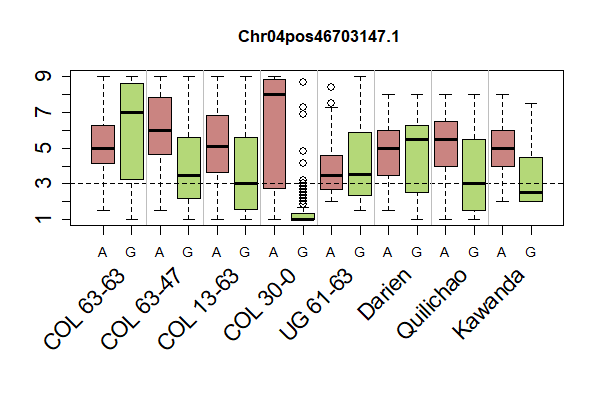 |
| ***Phg-2* on chromosome 8** | | |
| M1  Chr08pos61901182 | T/G | 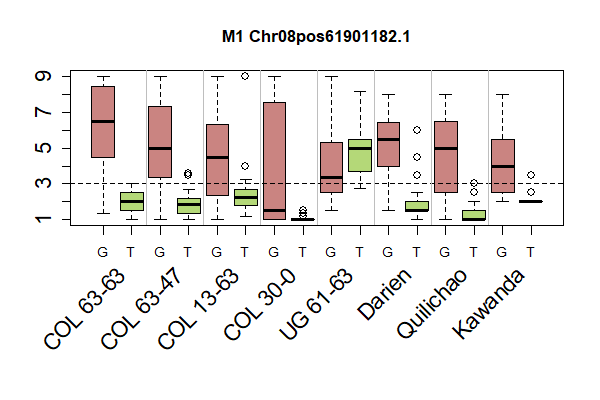 |
| M2  Chr08pos62188623 | T/C | 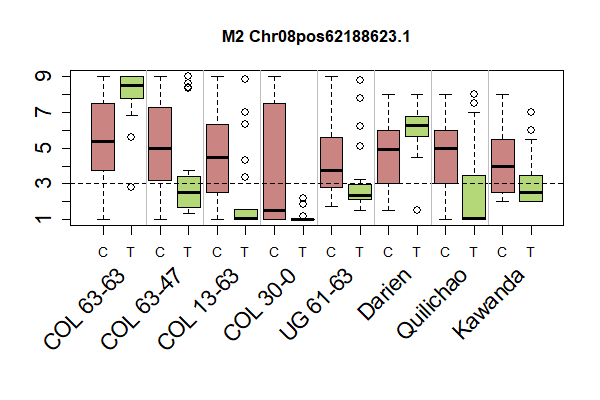 |
| M3  Chr08pos61828096  Chr08pos61878388  Chr08pos61880092 | C/A  A/C  T/C | 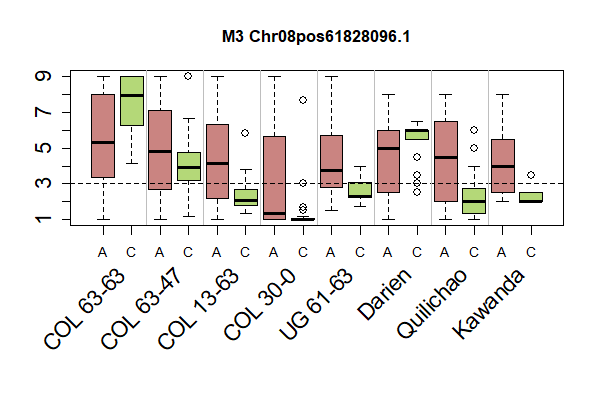 |
| M5 and M1^†^  Chr08pos61388457  Chr08pos61502023  Chr08pos61533289 | C/T  G/C  T/C | 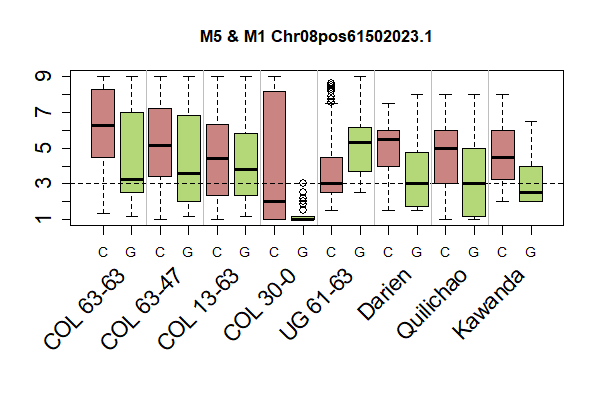 |
| A1  Chr08pos61825787  Chr08pos61879951  Chr08pos62191492 | A/C  C/A  G/A | 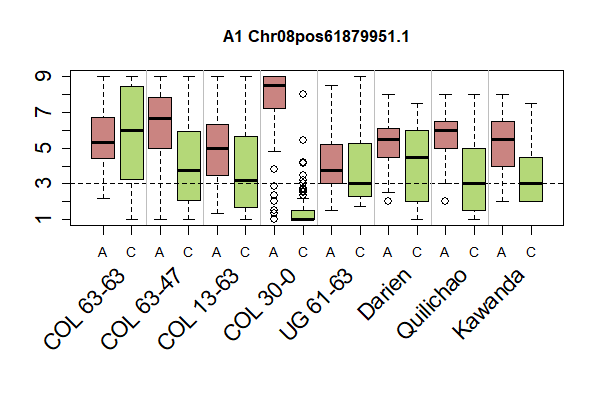 |
| A2  Chr08pos61828125 | G/A | 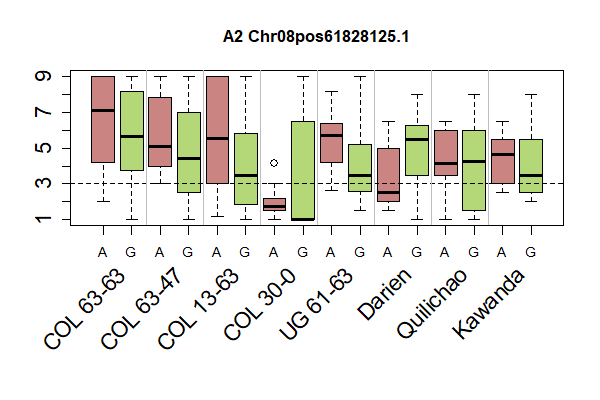 |
| A3  Not found |  |  |

^†^No SNPs that specifically tag M5 were found, but one that tags the
 haplotype group M5 and the highly resistant group M1 was found instead
